# The Interconnected Health Initiative: A Smithsonian Framework to Extend One Health Research and Education

**DOI:** 10.3389/fvets.2021.629410

**Published:** 2021-03-23

**Authors:** Pierre Comizzoli, Katrina M. Pagenkopp Lohan, Carly Muletz-Wolz, James Hassell, Brian Coyle

**Affiliations:** ^1^Smithsonian Conservation Biology Institute, National Zoological Park, Washington, DC, United States; ^2^Office of the Under-Secretary for Science and Research, Smithsonian Institution, Washington, DC, United States; ^3^Smithsonian Environmental Research Center, Edgewater, MD, United States

**Keywords:** One Health, disease, education, research, interdisciplinary, multidisciplinary, museum, collections

## Abstract

To better tackle diseases and sustain healthy ecosystems, One Health programs must efficiently bridge health in humans, domestic/livestock species, wild animals and plants, agriculture/aquaculture, and the environment. The Smithsonian Institution proposes to address this by considering ‘health' in a broad sense – the absence of undue pathogens and unnecessary stress for any organisms as well as access to good living conditions in functional environments. Considering the interconnectedness of all life forms, the Smithsonian plans to create a framework that will integrate cultural, social, and educational components into health research on humans, animals, plants, or ecosystems. The objectives of this perspective article are to (1) propose an innovative framework to support an interconnected/integrated approach to health and (2) provide examples fostering impactful collaborations on One Health research and education. Based on the core strengths of the Smithsonian (multidisciplinary research, outreach and education programs, libraries/archives, and collections) and central institutional support, this framework has the potential to extend existing health-related projects, address new needs and situations (e.g., response to pandemics), provide invaluable resources to inform policy and decision makers, and educate all audiences globally.

## Introduction

### Definitions of One Health and Examples of Current Programs

Historically, the concept of One Health has primarily focused on the spread of pathogens from animals to humans (zoonoses). However, the quality of the environment has major impacts on human health either directly through zoonotic disease transmissions or indirectly through management of natural resources (1). The World Health Organization (WHO) currently defines One Health as ‘*an approach to designing and implementing programs, policies, legislation and research in which multiple sectors, communicate and work together to achieve better public health outcomes*'. In the definitions of the United Nations Food and Agriculture Organization (FAO) the World Organization for Animal Health (OIE), or the Centers for Disease Control and Prevention (CDC), health outcomes depend on food safety, control of zoonoses, and combatting antibiotic resistance while recognizing the interconnection between people, animals, plants, and their shared environment. The One Health concept has now been extended beyond public health to include the ecological and environmental dynamics of disease in systems-based frameworks such as Planetary Health and Eco-Health (2–4). However, understanding the ecology and evolution of disease agents as well as defining health for humans, wildlife, ecosystems or food systems remains challenging because these processes are highly complex especially at a systems scale. Interactions among hosts, environments and disease agents also can be difficult to measure (3). Importantly, to tackle complex ecosystem and societal dynamics, One Health programs rely on strong collaborations with varying scopes and efforts [e.g., Eco Health Alliance www.ecohealthalliance.org; One Health Initiative www.onehealthinitiative.com; Lancet One Health Commission (5)]. Some programs are more centered on regional/global efforts and inform policy like the One Health Platform (https://onehealthplatform.com/home), or One Health Regional Network for the Horn of Africa (www.onehealthhorn.net). One of the Smithsonian's flagship programs in One Health is the Global Health Program which sits within the Conservation Biology Institute (www.nationalzoo.si.edu/global-health-program). Members of this team (clinical veterinarians, public health specialists, ecologists, and epidemiologists) have been studying health and disease at the human-animal interface since 2014. The scope is to improve global health conservation of wildlife species through (1) capacity building and training (2) research and (3) wildlife health. It has been a key coalition member of the USAID's Emerging Pandemic Threats Program (EPT) PREDICT project (www.usaid.gov/news-information/fact-sheets/emerging-pandemic-threats-program).

### More Actions Are Still Needed to Improve One Health

- A broader focus on all organisms and systems biology.

Beyond zoonotic diseases, the threat of non-infectious (including non-communicable) and infectious diseases of wildlife and plants must be considered. Additional threats include deforestation, urban expansion, unreasonable natural resource extractions, environmental pollution, stress, lack of fitness, invasive species, and climate change. A holistic view of interconnected life addressing these issues through different scales (from microbes to macro-organisms across local to global environments) is crucial for understanding the mechanisms by which health outcomes emerge from different environmental and anthropogenic states. However, this has not been fully achieved (6). For instance, invasive species are increasing due to globalization, environmental degradations and climate change. This leads to biodiversity loss and ecosystem impacts (e.g., pollinator decline, watershed degradation) that subsequently affect human health and livelihoods. More information is needed about co-infection dynamics within hosts and populations. More research also is required on how microbial infections impact host communities and change ecosystem functions (7). Many wildlife diseases have spread as a result of human activities (e.g., *Batrachochytrium dendrobatidis*, chytrid fungus in amphibians; *Pseudogymnoascus destructans*, white nose syndrome in bats; or *Canine morbillivirus*, canine distemper in wild carnivores). In these cases, the threat is not to humans directly, but among other species [e.g., chytrid fungus killing amphibians globally and decreasing food availability for snakes (8)].

- Incorporation of additional expertise in social/cultural and environmental science as well as museum collections.

Integration of environmental expertise, social science and behavioral aspects of health research and governance are limited or missing from many One Health programs (3). In that sense, some efforts have been made to integrate economic burdens of disease and social sciences into One Health programs (e.g., One Health Social Sciences Initiative of the One Health Commission www.onehealthcommission.org/en/programs/one_health_social_sciences_initiative/), but more could be done (9, 10). Similarly, while there is much research being done on climate and human health, more studies should be conducted on other forms of environmental change and health (e.g., land-use change) (11). In addition, efforts to improve health also must involve indigenous knowledge, local communities, governments, and educators. Importantly, socio-economic factors and societal inequities influence psychological health and risk of diseases [Chagas disease for instance (12)]. The current paradigm of limitless resource exploitation has to be replaced with more sustainable standards that better respect life on earth and strive toward well-being and social stability (13). Furthermore, more research and education are needed to address conflict resolution between stakeholders (14). Similarly, risk governance is required (irgc.org/about). Lastly, the One Health community should better leverage untapped research resources like natural history collections, libraries, and archives (15) while creating more biorepositories (16).

- Integration of formal (K-12 curriculum, undergraduate and graduate programs) and informal educational materials (exhibits, public programs, blogs).

As recommended by the National Academy of Medicine (17), it is crucial to define, develop, evaluate, improve, and continue to refine One Health education, not only in One Health degree programs but also in existing public health, environmental, agriculture, veterinary, and medical curricula. Besides improving scientific literacy of students, the general public also needs to be aware of and understand interconnections (humans are often part of the ecosystems) through museum exhibits and public programs.

Based on existing One Health definitions and current gaps, the objectives of this perspective article are to (1) propose an innovative framework to support an interconnected/integrated approach to health and (2) provide examples fostering impactful collaborations on Interconnected Health research and education (first at the Smithsonian and then in other organizations).

## Smithsonian Assets for a Novel Interconnected Health Framework

**Figure 1:**
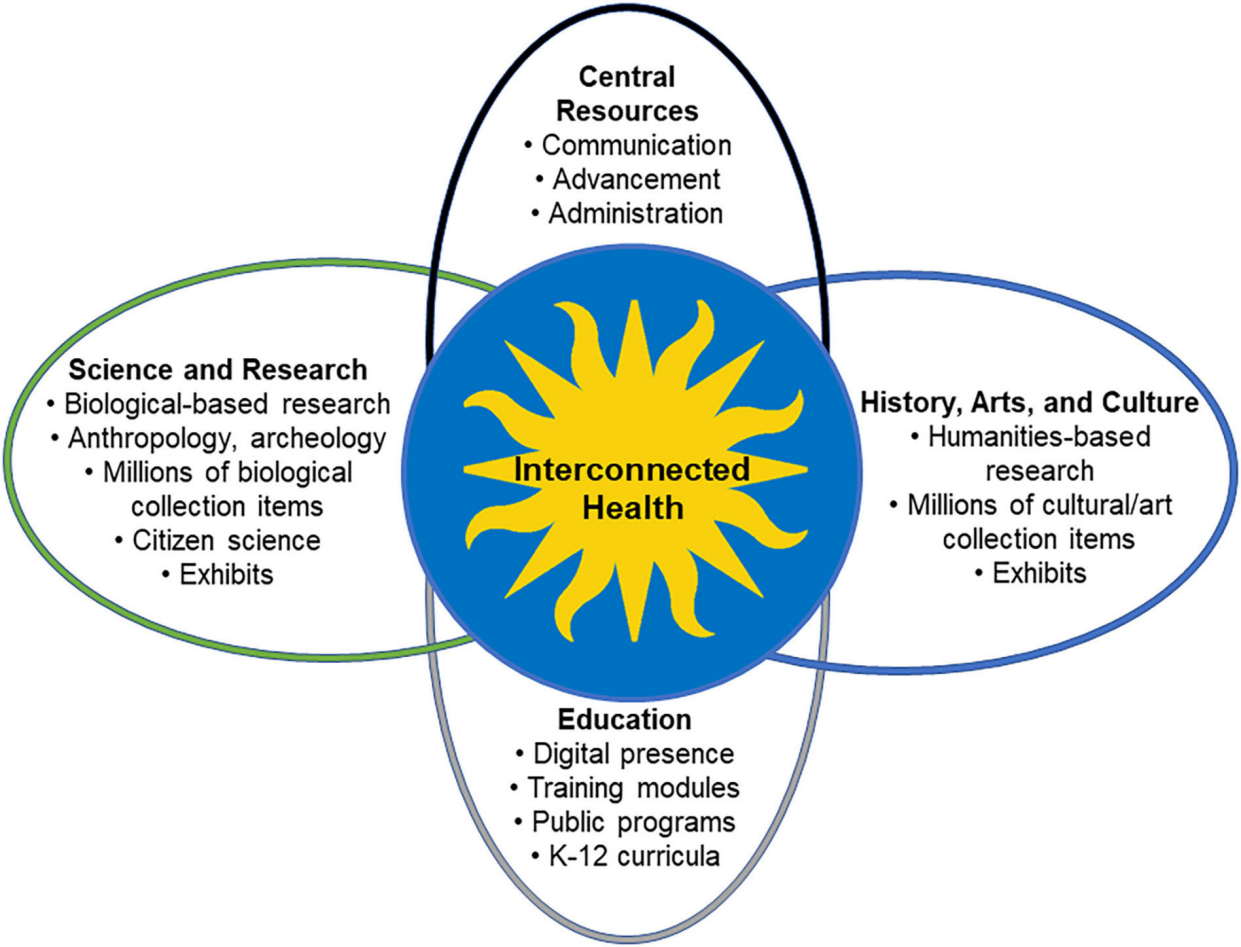
The new framework is at the interface of key Smithsonian resources and expertise.

Strong conservation infrastructure and broad disciplinary expertise are increasingly critical to supporting healthy ecosystems in the era of climate change and rapid degradation of biodiversity (18). An Interconnected Health framework is necessary to integrate multiple disciplines with a vision to improve health for all life through transdisciplinary research, education and rapid global action. A new framework will promote Smithsonian global leadership in One Health research and education. With 19 museums, a National Zoo, 14 education and research centers, libraries and archives, and one of the world's largest biological collections, the Smithsonian is poised to make a significant impact on health-related research and education (www.si.edu). Health is defined here in its broadest sense, as the absence of undue pathogens and unnecessary stressors for all organisms and the access to good living conditions in functional environments. This new framework that will support One Health research and education is at the interface of key resources and expertise across the institution (Figure 1). Here are some important details regarding the Smithsonian assets supporting the framework:

- Global presence across >140 countries and international projects (www.global.si.edu/) including research networks for forests (www.forestgeo.si.edu), marine environments (www.marinegeo.si.edu), and food systems (https://serc.si.edu/projects/oyster-reefs-and-restoration) as well as many long-term continuous research and monitoring programs across research centers and museums that started in the 20th and even 19th centuries.
- Training and education programs for students and professionals in health and disease: training in One Health and conservation medicine around the world (www.nationalzoo.si.edu/global-health-program), multidisciplinary fellowship programs in collaboration with many universities (www.smithsonianofi.com/fellowship-opportunities), practicum projects for students enrolled in Masters of Public Health, and curricula development (http://www.ssec.si.edu) (www.nmaahc.si.edu/learn/educators/stem-nmaahc).
- Large and growing citizen science programs (www.si.edu/volunteer/citizenscience) in research centers and museums (www.anacostia.si.edu/urbanwaterways/about).
- Excellence in exhibits and public engagement for tens of millions of visitors annually, in person and digitally: for example, “Outbreak: Epidemics in a Connected World” exhibit (and as a pop-up exhibit in 20 countries), “Modern Medicine and the Great War” or “Black Life in Two Pandemics” (www.naturalhistory.si.edu/exhibits/outbreak-epidemics-connected-world; www.americanhistory.si.edu/blog/black-life-two-pandemics).
- Infrastructure facilitating internal and external communication on health and disease through existing tools and programs: SI Profiles (www.profiles.si.edu)—a system that displays data from various sources, augmented by input from individuals, to showcase the depth and breadth of expertise at the Smithsonian; Conservation Commons (www.conservationcommons.si.edu)—a network combining Smithsonian cultural and scientific strengths in conservation.
- Hundreds of diverse partnerships across government agencies, non-governmental organizations, communities, and global networks of museums and science institutions.
- The Smithsonian has been offering world class research and public programming on pandemics for many years and has been addressing impacts of Covid 19 through many lenses including; K-12 curriculum; Outbreak! Epidemics in a Connected World exhibit; socio-cultural effects on communities of color; or the Pandemic Oral History project.

## Process and Methodology to Set up the Smithsonian Framework

Over the past 5 years, a series of meetings and discussions around the theme of Interconnected Health have involved more than 100 experts representing almost all Smithsonian units, offices, or programs. Based on ideas and information gathered during these consultations, a 5-year strategic road map was designed for implementing the framework.

The vision is that health of all life will be improved as a result of transdisciplinary research, engagement, and rapid global action. The mission of the framework is to advance knowledge and improve the health of all life globally through illuminating the interconnectedness of all organisms. To achieve the mission, three strategic objectives will integrate new components within and across projects to enhance reach and impact. Existing expertise will be leveraged to break down silos and encourage transdisciplinary thinking and collaboration; this will be done by assembling a workforce with expertise that spans the biological and social sciences, arts and humanities, and technology, with the capacity to implement pilot projects and grow programs.

The first strategic objective is to advance transdisciplinary research to understand how the interconnectedness of life forms and their environment affect the balance of health and disease in human impacted and natural systems (particularly including the humanities in these efforts). Success will be measured against several indicators, such as workforce diversity (across biological and social sciences, arts and humanities), use of novel technologies within individual research programs, or increase in interdisciplinary research outputs.

The second strategic objective is to develop formal and informal education/engagement plans that complement the Smithsonian's research programs and effectively convey the concept of interconnectedness. New knowledge generated by those two strategic objectives will be used to enhance existing health projects, make informed decisions leading to adapted action, catalyze protection of living organisms and habitats, and thereby support health globally. Success will be evaluated in the coming years by the increase in grant proposals and funded research integrating education and engagement.

A third objective will ensure programmatic and financial sustainability to support efforts in health research and education. In addition to leveraging and linking existing expertise to break down silos and encourage transdisciplinary thinking and collaboration, this will be done by assembling a workforce with the capacity to implement pilot projects and grow programs. A steering committee and an advisory committee will coordinate efforts across Smithsonian units to provide guidance in research and education. Core organizational structures will identify and assist with funding sources (grants, contracts, philanthropy) and communicate internally and externally while promoting equality, diversity and inclusion. Success of that objective will be achieved by the creation of a central office dedicated to Interconnected Health and the ability to secure large-scale support to interdisciplinary research conducted at national and global scales.

## How to Develop and Use the Framework?

**Figure 2:**
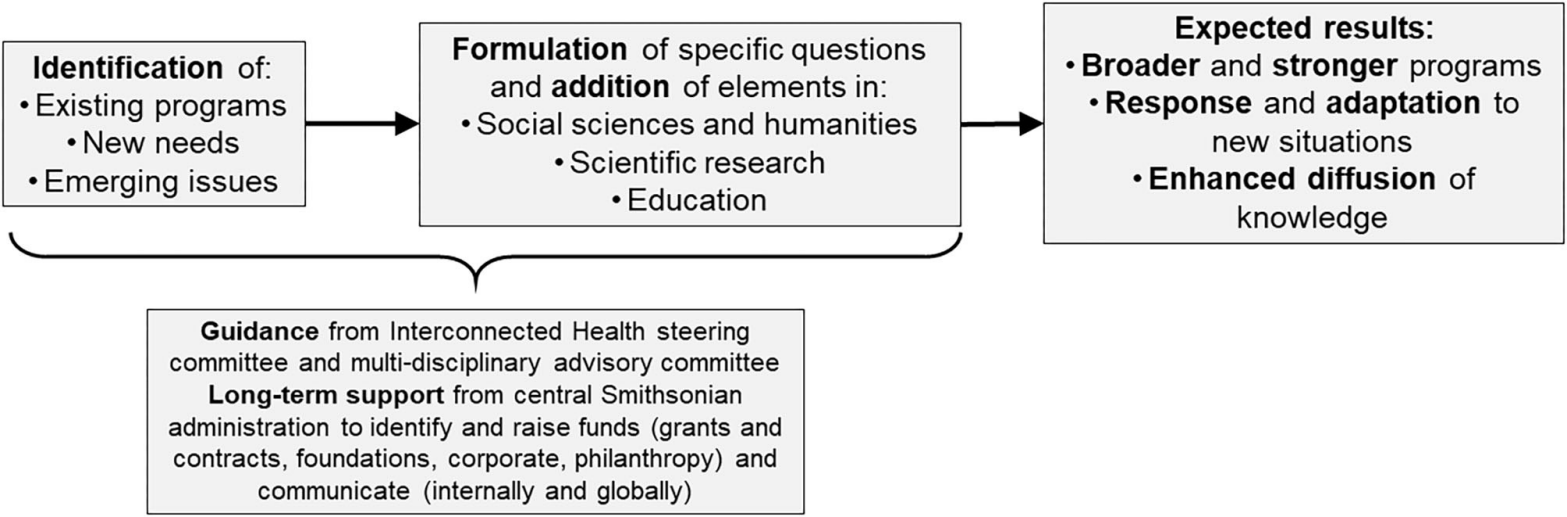
Flowchart illustrating the use of the Smithsonian framework to extend One Health research and education.

Based on the strategic objectives mentioned above, new components will be integrated within and across projects. A network of interdisciplinary practitioners will also be used to identify priorities for research and education to better prioritize and guide activities. Once existing programs, new needs, or emerging issues are identified, the use of the framework will be driven by sound questions in science, humanities and education (Figure 2). For instance:

- How can Smithsonian experts work more effectively across disciplines to better understand pathogen spillover into new host species and environmental pollution, and to mitigate disease impacts through health monitoring, habitat conservation, public education, and cultural knowledge?
- How do we measure the value of, and best conserve, vital ecosystem services in human impacted areas through science, community-engagement and education?
- How can we understand and mitigate the impacts of social inequity and inequality on human physiological and psychological health as well as environmental and wildlife health?

While specific questions are being formulated, key elements will be integrated into existing programs, new needs, or emerging issues (Figure 2). For instance:

- Providing unique expertise (cultural aspects) and professional training opportunities to ongoing One Health programs.
- Promoting community engagement, public education, and preparedness.
- Creating unified messaging and education that is shared by all museums to promote interconnected/integrated approach to health among the general public and in schools.

## Expected Results of the Framework

### Building Broader and Stronger Programs Upon Existing Smithsonian Projects

While the microbial disease component is crucial and already present in several Smithsonian projects, progress is being made in incorporating a focus on how to keep microbiota functional and healthy in agricultural and forest systems, conservation breeding, and reintroduction efforts. The Smithsonian also has microbe-related education projects (Outbreak exhibit) and new cultural projects (American Stories in the time of Pandemic, Art in Quarantine). However, the new framework is not solely centered on micro-organisms. Here are some examples of innovative programs within and across current activities that will help achieve a broadly integrative and holistic approach:

- Community/Species-based programs

Health and conservation of amphibians (www.nationalzoo.si.edu/center-for-species-survival/panama-amphibian-rescue-and-conservation-project) or coral reefs (www.ocean.si.edu/ecosystems/coral-reefs) are good examples that easily fit within the new framework. Both programs conduct and translate research for education and to inform policy. In the Urban Waterways Project, local communities are engaged with their environment through a holistic approach that incorporates science, art, education, religion, and the humanities (www.anacostia.si.edu/urbanwaterways/about).

- Land and seascape-based programs

Agua Salud develops approaches for tropical forest management (www.striresearch.si.edu/smartreforestation) to the mutual benefit of communities and nature. Similarly, working land and seascapes (www.wls.si.edu) aims to foster healthy and productive landscapes and seascapes. The Global Change Research Wetland houses several long-term experiments to unravel the complex ecological processes that occur in coastal wetlands and how climate change is impacting them (www.serc.si.edu/gcrew). Regarding sustainable food systems, the Marine Conservation Program combines social, ecological and economic research to protect and improve the health of marine systems that support fisheries and communities (global.si.edu/success-stories/see-how-smithsonian-working-local-fishermen-protect-marine-areas-and-create-safe). Smithsonian collections of over 35 million insect specimens have been an invaluable resource to agriculture science and management since the 19th century (agresearchmag.ars.usda.gov/2002/jun/bugs). With partners in East Africa and Asia, the Global Health Program (https://nationalzoo.si.edu/global-health-program) is studying how environmental and demographic pressures that push people and their livestock into closer contact with wildlife, create opportunities for the emergence and spread of novel diseases in people and animals. Landscape-scale experiments are being conducted in natural ecosystems, inhabited by people and their livestock, and cities, where rapid urban development—characteristic of cities in the tropics—can bring wildlife and livestock into close contact.

- Region-based programs (multiple landscapes and ecosystems)

The Mpala Research Center, in Laikipia, Kenya (www.mpala.org) —of which the Smithsonian is a founding member—provides a ‘living rangeland laboratory' in which Smithsonian and international researchers conduct landscape-level experiments and training on ecology and health (e.g., wildlife endocrinology, emerging zoonotic disease, landscape connectivity, nutrient cycling), translating their findings into solutions for wildlife conservation, sustainable livelihoods and ecosystem resilience. Across land and seascapes, Movement of Life (www.movementoflife.si.edu) advances the understanding of how all living things, big and small, move across changing land and seascapes.

Note that all of programs mentioned above integrate the well-being of humans and wildlife, economics, cultural heritage, national and global biosecurity, exhibits and public programs, and curriculum for schools.

### Respond and Adapt to New Situations

A recent call for a COVID-19 One Health Research Coalition is a good illustration about the need for multidisciplinary and multilateral coalitions (19). The framework currently is helping the Smithsonian response to the COVID pandemic in research, humanities, museum exhibit and education (facilitating internal communication, enhancing exploration of museum collections, improving the search for research funds, initiating new programs). Lessons learned will also inform new pan-institutional efforts tackling global issues. Through the implementation of this framework, the Smithsonian would be well-placed to respond to future planetary challenges that threaten human, environmental and animal health.

### Enhance the Diffusion of Knowledge

Smithsonian exhibits and education programs can easily incorporate new material and generate new content for public education (e.g., COVID-19 module from the Science Education Center). The Smithsonian's position in the US government provides important opportunities to participate in congressional hearings and interagency committees (including NIH, USAID, USDA, CDC) that vitally inform policy making. Many government leaders attend Smithsonian programs or visit exhibits and meet with scholars to learn about important issues. In addition to interfacing science/culture with American and foreign policy, the global reach of its staff (many of whom work in partnership with foreign governments, local institutions, and communities around the world) will help identify new and important questions for research and help design innovative educational approaches for higher impact.

## Conclusions

The Smithsonian interconnected approach to health integrates science, culture and education. It allows to involve more experts, find innovative solutions, engage a wider audience, and increase impact. Although the proposed framework is specific to the Smithsonian, lessons learned (in programmatic and financial sustainability) and corrective solutions will be shared with the community to foster similar approaches in other organizations. Importantly, the approach has the possibility to change attitudes across societies and facilitate intergenerational dialogues on a global scale about vital issues. It also inspires a next generation of scientists who will diversify STEM fields and stimulate creative expertise in health and disease related research. This integrative approach should be added to the professional development of practitioners and educators. Lastly, the framework will address and help increase scientific literacy, diversity, equity, accessibility, and inclusion.

## Data Availability Statement

The original contributions presented in the study are included in the article/supplementary material, further inquiries can be directed to the corresponding author/s.

## Author Contributions

PC, KP, CM-W, JH, and BC contributed equally to the collection of information, outline of the manuscript, and writing. All authors contributed to the article and approved the submitted version.

## Conflict of Interest

The authors declare that the research was conducted in the absence of any commercial or financial relationships that could be construed as a potential conflict of interest.
